# The Liver and Its Diseases

**Published:** 1853-01

**Authors:** 


### ECLECTIC DEPARTMENT,

### AND SPIRIT OF THE MEDICAL PERIODICAL PRESS.

---

*The Liver and its Diseases.* By W. B. Herrick, M. D., Chicago, Illinois.

> "The jaundiced thus see all things round them clad
> In yellow; every object as it flows
> Meeting new tides of yellow, from their forms
> Thrown forth incessant; and the lurid eye,
> Deep, too, imbued with its contagious hue,
> Painting each image that its orb assails."

The above quotation from Lucretius, descriptive of a class of persons whose defective visual organs "see all things round them clad in yellow," cannot fail to remind the reader of certain practitioners, the patients of whom are always *bilious.*

With them constipation or diarrhœa, dry skin or profuse perspiration, want of sensibility or extreme irritability, alike indicate that their patients are bilious, and require, therefore, in their treatment, blue pill, calomel, or some other mercurial.

This class of physicians, who thus make diseases so unlike in character and symptoms dependent upon the same cause, and, as a consequence, adopt the routine practice above indicated, must be deficient in judgment and mental capacity; or, what is worse, too indolent to obtain and appropriate to their use the facts and information acquired by others, by which their mental vision might be extended, so as to embrace more than a single class of diseases and one mode of treatment.

In order to show that we are fully justified in making these strictures upon this class of practitioners, we will state briefly what is now known of the structure and functions of that organ, upon the abnormal condition of which these so-called bilious affections are supposed to be dependent.

The *liver*, as is well known, is a glandular organ, constituted of cells, excretory ducts, and blood-vessels. The cells are supplied by the vena portarum with the imperfectly elaborated and impure venous blood, directly from the absorbing mucous surfaces of the stomach and intestines; whilst the ducts, on the other hand, are surrounded by the terminal branches of the hepatic artery, containing pure blood from the great arterial current.

From recent physiological investigations, it appears highly probable that the hepatic cells abstract from the impure blood in the portal vein the starchy, and perhaps some other carbonaceous substances derived from food, and change them either into the fatty constituents of bile, or into sugar, to be reabsorbed by the hepatic veins.

That this change from starch granules to fat globules does in reality take place in the hepatic cellc of the higher order of animals, is rendered almost certain by the observations made by Liedy upon the follicular liver of the crustacea.

"When," says he, "a cæcum, is viewed beneath the microscope, its lower half appears filled with a finely granular matter, and the anterior half with a mass of fat cells." That some of the carbonaceous substances contained in the blood are changed into sugar, during its passage through the liver, is made evident by the recent very conclusive and highly philosophical investigations of M. Bernard.

"He examined," says Donaldson, "the contents of all the principal venous trunks: the vena porta, the inferior and superior cava, the jugular, &c., and, singular to say, he could nowhere detect its presence, (sugar,) but in the hepatic veins, and in the ascending cava, and thence to the right auricle. There being no trace of it in the blood flowing into the liver, nor yet in the pulmonary veins, was not our experimenter justified in coming to the conclusion that it was fabricated in the liver and destroyed in the lungs?"

According to Liebig, the saccharine constituents of blood are, by two successive stages of oxidation, converted primarily into lactic acid, and finally into carbonic acid and water. Hence it would appear that sugar, whether absorbed directly as such, or formed in the liver, in the manner above indicated, supplies by its combustion the amount of animal heat required over and above that which would necessarily result from other and more important chemico-vital changes.

In view of these facts, it is rendered highly probable, if not absolutely certain, that the office of the hepatic cells is to take up the starchy materials, contained in the portal blood, and convert them either into fat or sugar, according as they are required or not to subserve the immediate purposes of respiration—into sugar when, from a deficiency of lactic acid and other organic compounds readily convertible into carbonic acid and water, there is a deficiency; and into fat, when an excess of these substances affords already an abundant supply of respiratory food.

The sugar thus formed is taken up by the hepatic veins, and passes immediately into the circulation, there to be changed by oxydation; first into lactic or some other organic acid, and finally into carbonic acid and water.

The fat, on the other hand, passes into the terminal branches of the hepatic ducts, where it finds, in the capillary net-work derived from the hepatic arteries by which they are surrounded, an abundant supply of arterial blood. This, doubtless, furnishes both the oxygen and the alkali, by which the fatty matter is rendered soluble, and made to pass readily and easily through the small hepatic ducts as a fatty acid combined with soda, in the form of bile.

These views of the physiological action of the liver are fully sustained by numerous facts, physiological, pathological, and chemical, which, however, cannot be presented in the short space allotted to this article; it being our object at this time, not to sustain our own peculiar physiological views, but to make such practical suggestions as may serve to direct the attention of our readers to the subject, and to show them the absurdity of the present indiscriminate mode of practice, adopted by many, in the so-called bilious affections, supposed to be dependent always upon some morbid condition or action of this much-abused organ.

From what has been said, it is evident that in warm latitudes, and in summer, when there is less oxygen, and, consequently, more lactic and other organic acids in the blood, the liver must change a larger proportion of the starchy constituents of food into fat. If the amount of oxygen and free soda in the blood is sufficient to combine with this fat, and render it soluble, it passes readily out of the liver into the intestines, in the form of bile, and is reabsorbed by the lacteals, like other fatty matter, and no indications of disease appear; or if in great excess, it passes off in the form of profuse bilious discharges, so common in the summer, especially in the South and West. A still greater deficiency of oxygen, and consequent accumulation of organic acids in the blood, to combine with its alkaline constituents, would diminish proportionally the amount of free soda, and thus prevent it from entering into the constitution of bile to a sufficient extent to make it perfectly soluble, and to neutralize its fatty acids, and thus give rise to acrid and vitiated bilious discharges, or to congestion, torpidity and enlargement of the liver, from an accumulation of imperfectly dissolved fatty matter in the hepatic ducts.

Admitting the correctness of the above views, it is evident that the proper treatment of the whole class of liver affections, above enumerated, would be the administration of alkalies, especially those which are among the natural constituents of blood, such as potash and soda.

Two years' experience in the use of potash and soda, in some of their forms, as remedies in the above named class of diseases, has convinced the writer that one or both may be used with confidence as substitutes for calomel in the treatment of such cases.

That the class of remedies under consideration was formerly used much more extensively than at present in liver affections, is evident from the following quotation from Good's Study of Medicine, published in 1829, in which, after discussing the merits of the dandelion as a remedy for jaundice, the author remarks, that "soap and alkalies seem to have much better pretensions to favor, and have been still more widely employed in this disease, and pretty generally regarded as general, and hence hepatic solvents.—*Northwestern Med. and Surg. Journal.*

